# Dietary carbohydrate structure affects the gut microbial diversity, succession, and compartmentalization in black soldier fly larvae

**DOI:** 10.3389/fmicb.2026.1956925

**Published:** 2026-10-29

**Authors:** Shaojuan Liu, Yangjie Lu, Xiaotian Tang, Yancheng Liu, Xiaowei Pan, Shaodong Zeng, Jianzhi Ye, Hongbin Guo, Huifang Ma

**Affiliations:** 1 Laboratory of Quality & Safety Risk Assessment on Agro-Products Processing, Ministry of Agriculture and Rural Affairs, Agricultural Products Processing Research Institute, Chinese Academy of Tropical Agricultural Sciences, Zhanjiang, China; 2 Universiti Malaya, Kuala Lumpur, Malaysia

**Keywords:** 16S rRNA sequencing, black soldier fly larvae, dietary carbohydrate, gut microbiota, waste bioconversion

## Abstract

**Introduction:**

Black soldier fly larvae (BSFL) plays a pivotal role in bioconverting organic wastes into high-value biomass by gut microbiota. However, how dietary carbohydrate structure modulates gut microbial assembly remains unclear.

**Methods:**

Here, we characterized the gut bacterial communities across developmental stages and gut regions of BSFL reared on wheat bran (fiber-rich) and cassava (starch-rich) diets using 16S rRNA sequencing. Alpha-diversity analysis revealed that wheat bran diet significantly enhanced species richness (ACE, Chao) and diversity (Shannon), with progressive increases across larval stages, whereas cassava diet only increased richness without improved evenness. Dietary substrate was the primary driver of gut microbiota composition (PC1: 32.5% variance), followed by developmental stage. Proteobacteria, Firmicutes, Bacteroidota, and Actinobacteriota were the predominant bacterial phyla across both diets. Firmicutes dominated the wheat bran group (52.34%) with a successional shift from Proteobacteria-enriched early stages to Firmicutes-enriched later stages, while Proteobacteria prevailed in cassava group (79.27%) throughout development. The wheat bran group was dominated by the genera of *Klebsiella* (20.17%), *Bacillus* (18.17%), *Providencia* (10.15%), whereas cassava group by *unclassified_f_Enterobacteriaceae*, *Klebsiella*, *Enterococcus*, *Providencia*, and *Morganella*. Specially, Bacillaceae prevailed in the anterior gut and the hindgut and Lachnospiraceae in the hindgut under wheat bran diet, indicating sequential fiber degradation. Cassava diet exhibited a homogeneous Enterobacteriaceae dominance across all regions. LEfSe further identified Firmicutes-affiliated taxa enriched in wheat bran and Dysgonomonas-associated taxa in cassava.

**Discussion:**

Collectively, these findings demonstrate that dietary carbohydrate structure dictates the taxonomic composition of the BSFL gut microbiota, offering practical guidance for optimizing waste bioconversion stratˇegies based on feedstock characteristics.

## Graphical abstract

**Figure:**
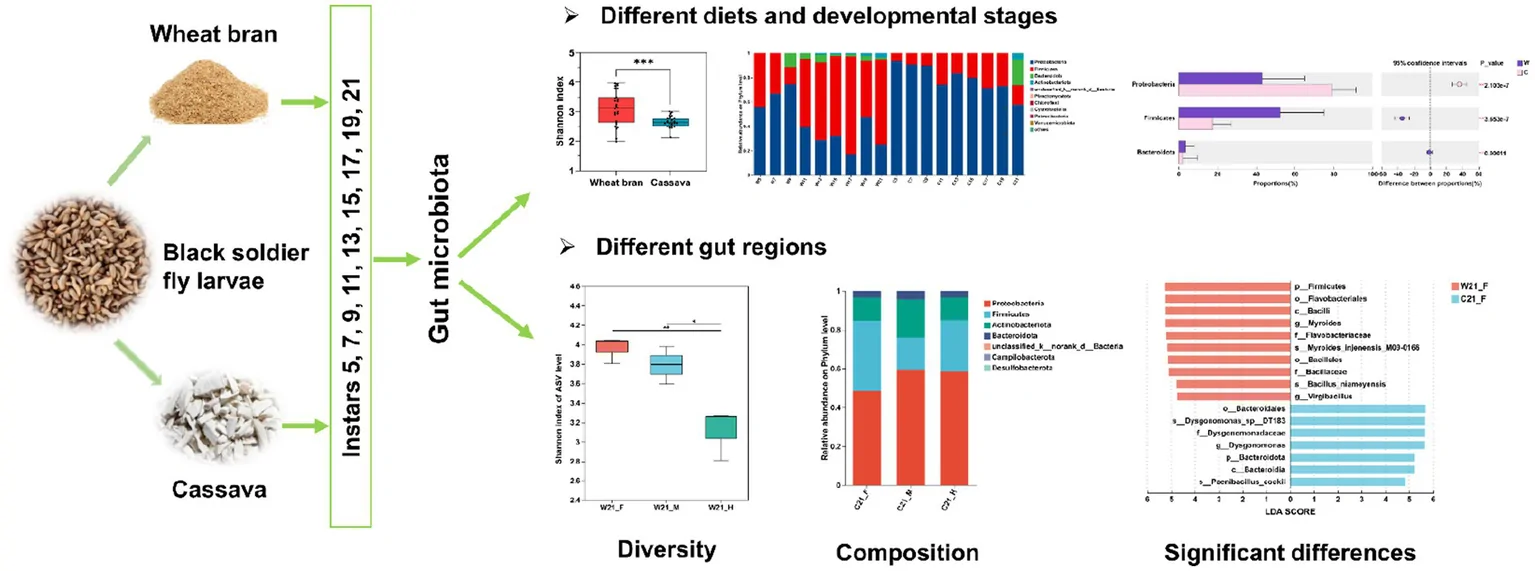

## 1 Introduction

Black soldier fly larvae (BSFL, *Hermetia illucens* L.) have emerged as a promising agent for organic waste management, capable of efficiently converting a wide range of biodegradable substrates into nutrient-rich larval biomass and suitable for animal feed and biofuel production (Müller et al., 2017; Ungureanu and Vladut, 2026). Fruit and vegetable wastes typically contain 40%–60% non-fibre carbohydrates (NFC) on a dry matter basis, predominantly starch and soluble sugars (Chapagaee et al., 2026; Cohn et al., 2022), whereas agricultural residues such as wheat straw and rice straw contain 50%–70% fibre, including 22.1%–30.3% cellulose, 15%–22.1% hemicellulose, and 8.0%–15% lignin (Abduh et al., 2021). However, the higher lipid accumulation in starch-fed BSFL may represent a trade-off in bioconversion performance. Specifically, starch-rich substrates promote *de novo* lipogenesis from excess carbohydrates, resulting in increased crude fat content but reduced relative protein proportion (Eggink et al., 2023; Schøn et al., 2026). Since BSFL are primarily valued as a protein source for animal feed, excessive lipid deposition at the expense of protein synthesis may diminish the nutritional quality of the harvested biomass. The bioconversion capacity of BSFL is intimately linked to their gut microbiota, which supplies essential digestive enzymes and facilitates nutrient assimilation (Wang et al., 2024). The diversity and structure of microbial communities in insects vary at different stages of biological processes (Malacrinò, 2022; Yun et al., 2014). Understanding the factors that shape the assembly and function of the larval gut microbiome is therefore of both fundamental ecological significance and applied biotechnological value.

Accumulating evidence has demonstrated that dietary composition is a primary determinant of gut microbial community structure in insects, while the developmental stage only affects the microbial community of the entire individual (Huang et al., 2021; Wang et al., 2020). A study has compared gut microbiota of BSFL across various feedstocks, including fruits, vegetables, grains, and animal manures, revealing substrate-specific microbial signatures (Gurusinga et al., 2026). For instance, using flaxseed meal, tiger nut meal and pepper seed meal as feeding substrates for BSFL, Firmicutes were found to be the dominant bacteria in these four substrates, whereas their relative abundances were different (Wang et al., 2025). The result showed that *Sedimentibacter*, *Clostridium*, *Enterococcus*, *Bacteroides* and *Bacillus* were significantly enriched in the BSFL gut fed on sericulture waste, while there were higher abundances of potential pathogenic and harmful bacterial genera in the unfermented sericulture waste group, including *Providencia*, *Klebsiella*, *Escherichia* and *Brucella* (Memon et al., 2025). BSFL gut microbial community shows differences among different intestinal segments that the microbial diversity in the anterior intestine is the highest, gradually decreasing along the mid-intestinal direction, while the bacterial load shows the opposite trend, being the highest in the posterior intestinal region (Auger et al., 2023). The study has been reported that Proteobacteria, Firmicutes, Bacteroidota and Actinobacteria were consistently the dominant microbiota across developmental cycle and dietary Housefly Gainesville and poultry hatchery waste (Auger et al., 2023). Consistently, Li et al. (2023) revealed that Actinobacteria, Proteobacteria, Firmicutes, and Proteobacteria were the most dominant bacterial phyla across all larval stages of BSF gut, while the bacterial community composition in different larval stages were significantly varied at the genus level. Meanwhile, it revealed that the intestinal bacteria promote the growth and development of BSF. The nutritional profile of the feeding substrate particularly its carbohydrate composition can profoundly influence the growth performance of BSFL, waste reduction efficiency, and the final nutritional quality of harvested biomass (Shelomi, 2020). However, most previous studies have focused on comparing overall microbial diversity across different waste types, or a little attention to the dynamic processes of community assembly over developmental time and along the spatial axis of the gut. The insect gut is functionally compartmentalized, and dietary carbohydrate structure may dictate whether digestion is spatially stratified (fiber) or centralized (starch).

The insect gut plays a crucial role in the degradation of organic matter by its intestinal microbiota, including various difficult-to-degrade substances such as lignin and polysaccharides (Li et al., 2017; Yang et al., 2018). Similarly, BSFL can also degrade anti-nutritional factors such as lignin and cellulose through intestinal microbiota (Asano et al., 2022; Klüber et al., 2022). Carbohydrates are the main source of energy in the diet of BSFL. A study has shown that soluble carbohydrate diet can significantly affect the growth performance and fatty acid profiles of BSFL when compared with a cellulose diet, whereas the role of the microbial community is worthy of exploration (Carpentier et al., 2024). Consistently, in our previous research, it has indicated that cassava-fed BSFL has the highest crude fat content when compared to the diets of wheat bran, peanut cake, soybean meal cake, and corn distillers’ grains (unpublished data). Wheat bran and cassava represent two nutritionally distinct substrates with contrasting carbohydrate compositions. Wheat bran is rich in complex dietary fiber (approximately 40–50% dry matter), including cellulose, hemicellulose, and lignin, which are characterized by β-1,4 glycosidic bonds and extensive cross-linkages that require synergistic microbial enzymatic activities for complete degradation (Saini et al., 2023). Cassava, in contrast, is predominantly composed of easily digestible starch (approximately 70–80% dry matter), a glucose polymer with α-1,4 and α-1,6 linkages that can be rapidly hydrolyzed by amylases (Chamorro et al., 2025). Organic wastes destined for BSFL bioconversion vary widely in carbohydrate composition. For example, food waste typically contains 13–15% non-fibre carbohydrates (NFC, primarily starch and soluble sugars) on a dry mass basis (Gold et al., 2020), with total carbohydrate contents reaching 40–60% in some streams (Cohn et al., 2022). In contrast, agricultural residues such as wheat straw and bran are rich in structural fibre, with fiber contents ranging from 50% to 70% (Chapagaee et al., 2026; Xu et al., 2025). This fundamental difference in carbohydrate composition readily fermentable NFC versus recalcitrant structural fibre may drive distinct patterns of microbial assembly and degradation in the BSFL gut.

In this study, we conducted a comprehensive 16S rRNA sequencing analysis to characterize the gut bacterial communities of BSFL reared on wheat bran and cassava diets across developmental stages and gut regions. Specifically, we aimed to (1) To compare the diversity and composition of gut microbiota between wheat bran diet (fiber-rich) and cassava diet (starch-rich diets); (2) To delineate the temporal succession patterns during larval development; (3) To determine whether dietary carbohydrate structure modulates spatial organization along the gut axis; and (4) To identify substrate-specific and region-specific bacterial taxa as potential functional markers. The findings of this study will provide an understanding of the interaction of dietary carbohydrates and BSFL gut microbiota, offer the rational selection of waste substrates for optimized bioconversion outcomes, and predict potential biosafety risks.

## 2 Materials and methods

### 2.1 BSFL feeding

The BSFL eggs were provided by Sichuan Yangyun Biotechnology Co., Ltd. After incubation on wheat bran for 3 days, BSFL were reared on the diets of wheat bran and cassava, separately. Both wheat bran and cassava diets were screened through a 12-mesh sieve. The nutritional components of wheat bran diet and cassava diet are shown in Supplementary Table S1. For each dietary treatment, three independent plastic containers (25 × 16 × 8 cm) were prepared, each containing larvae hatched by 0.20 ± 0.01 g BSFL eggs. The containers were maintained under identical conditions (27 °C ± 0.5 °C, 70% relative humidity). Each container served as an independent biological replicate, and all sampling was performed separately for each container. Thus, *n* = 3 biological replicates per diet per time point. All containers were surface-sterilized with 75% ethanol and air-dried before use. The rearing room was maintained under positive pressure with HEPA-filtered air to minimize airborne contamination. They were raised for 21 days. During the entire larval stage, samples were collected at the 9th instar (5th, 7th, 9th, 11th, 13th, 15th, 17th, 19th, and 21st instars) and stored at −80 °C for subsequent analysis.

### 2.2 Collection of intestine

The larvae of different instars were collected and then starved for 24 h to empty their feed and digestive contents. For each dietary group and each time point, 10 larvae were collected. For whole-gut samples, 5 larvae were randomly selected and their entire guts were pooled as one biological replicate; this was repeated to obtain 3 biological replicates per time point per diet. For gut-region samples (21 d only), 15 larvae per diet were dissected into foregut, midgut, and hindgut. For each region, 5 guts were pooled as one biological replicate, with 3 biological replicates per region per diet. The surface impurities of larvae were, respectively, washed off with sterile water, 75% ethanol and enzyme-free water for three times, each time for 3 min. Then, the larvae were thoroughly rinsed with sterile water and dried with paper. Finally, the BSFL guts were collected to 1.5 mL sterile centrifuge tube by cutting the abdomen using sterilized tweezers and scissors in a sterile environment. The gut samples were quickly frozen with liquid nitrogen and stored at −80 °C for the analysis of gut bacteria.

### 2.3 DNA extraction

The genomic DNA of BSFL gut bacteria was extracted using E.Z.N.A.® soil DNA kit (Omega Bio-tek, Norcross, GA, U.S.) according to manufacturer’s instructions. The quality and concentration of DNA were determined by 1.0% agarose gel electrophoresis and a NanoDrop 2000 spectrophotometer (Thermo Scientific, United States) and kept at −80 °C for sequent sequencing.

### 2.4 16S ribosomal RNA (rRNA) gene sequencing

The hypervariable region V3–V4 of the bacterial 16S rRNA gene were amplified with primer pairs 338F (5′-ACTCCTACGGGAGGCAGCAG-3′) and 806R (5′-GGACTACHVGGGTWTCTAAT-3′). The sequencing was conducted on an Illumina Nextseq2000 platform (Illumina, San Diego, USA). The Illumina Nextseq2000 platform generated 2 × 250 bp paired-end reads, which were merged to obtain V3–V4 amplicons of approximately 460 bp. The raw data was quality filtered with fastp (v 0.19.6). The high-quality sequences were de-noised using DADA2 plugin in the Qiime2 (v 2024) and then the amplicon sequence variants (ASVs) were obtained. To minimize the effects of sequencing depth on diversity measures, all samples were rarefied to 20,000 sequences per sample, which yielded an average Good’s coverage of 97.90%. Rarefaction curves confirmed that this depth was sufficient to capture the majority of bacterial diversity. Taxonomic assignment of ASVs was performed using the Naive bayes consensus taxonomy classifier implemented in Qiime2 and the SILVA 16S rRNA database (v138). Based on the ASVs information, rarefaction curves and alpha diversity indices including Chao1 richness, Shannon index, and ACE index were calculated with Mothur (v1.30.1). Alpha diversity indices (ACE, Chao1, Shannon) were compared among groups using one-way ANOVA followed by Tukey’s HSD when data met normality (Shapiro–Wilk test) and homoscedasticity (Levene’s test) assumptions; otherwise, Kruskal–Wallis H followed by Dunn’s test was used. The similarity among the microbial communities in different samples was determined by principal coordinate analysis (PCoA) based on Bray–curtis dissimilarity using Vegan v2.5–3 package. Beta diversity was assessed using Bray-Curtis distances, and PCoA was performed for visualization. PERMANOVA (Adonis) with 999 permutations was used to test group differences, and PERMDISP was used to check homogeneity of dispersions. For differential abundance analysis, relative abundance data were not transformed; Kruskal-Wallis H test (*p* < 0.05) was used for inter-region comparisons within each diet, and LEfSe (LDA > 4, *p* < 0.05) was used for inter-diet comparisons within each gut region. The variance inflation factor (VIF) for each variable was estimated using the vif function in the car package.

### 2.5 Statistical analysis

All statistical analyses were performed on 16S rRNA gene sequencing data. No non-microbiome data were generated in this study. Data illustration and analysis were performed by GraphPad prism 8.0 software. The results presented means ± standard deviations, with a minimum of three biological replicates. One-way analysis of variance (ANOVA) and *t*-test were used to determine the differences among samples from multi-groups with significance levels of *p* < 0.05.

## 3 Results

### 3.1 Diversity of intestinal bacterial communities in BSFL

After quality filtering, a total of 2,617,650 clean sequences were obtained, clustered into 3,226 ASVs across all samples. The rank-abundance curve revealed that the wheat bran group exhibited a longer and smoother curve than the cassava group (Supplementary Figure S1), indicating higher species richness and community evenness under the fiber-rich diet. Alpha-diversity analysis confirmed that the wheat bran group had significantly higher ACE, Chao, and Shannon indices than the cassava group (Figures 1A–C). When examining temporal dynamics within each diet, distinct patterns emerged. In the wheat bran group, all three diversity indices increased significantly with larval instar (Figures 1D–F). In contrast, in the cassava group, only the richness estimators (ACE and Chao) were significantly higher at instar 21 than at instar 7, while the Shannon index showed no significant change across instars (Figures 1G–I). These results indicate that the wheat bran diet supported a more species-rich and even gut microbial community than the cassava diet.

**Figure 1:**
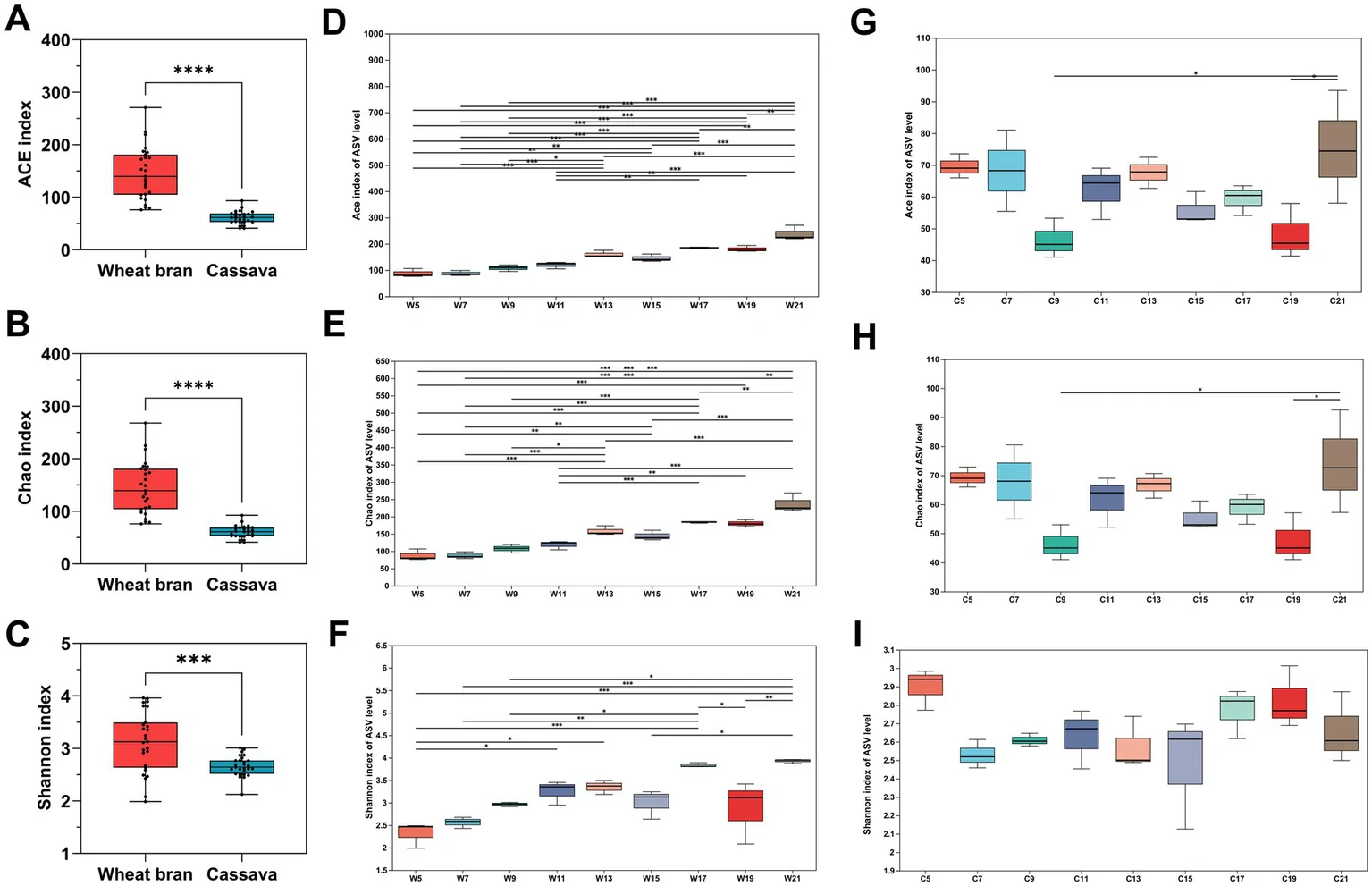
Alpha diversity of the intestinal bacterial microbiota in BSFL. **(A–C)** ACE, Chao1, and Shannon indices comparing the wheat bran and cassava diets. **(D–F)** ACE, Chao1, and Shannon indices across larval instars within the wheat bran diet group; **(G–I)** corresponding indices across larval instars within the cassava diet group.

To compare the structural dissimilarity of larval gut bacterial communities across substrates and instars, we performed principal coordinate analysis (PCoA) based on Bray-Curtis distances. The PCoA plot revealed a clear separation between wheat bran- and cassava-fed larvae along PC1 (32.5% variance), indicating that dietary substrate was the dominant factor shaping gut community structure (Figure 2A). Within the wheat bran group, larvae at instars 5–9 were clearly separated from those at instars 11–21 along PC1 (41.39% variance), with instars 11–15 and 17–21 forming two distinct subclusters (Supplementary Figure S2A). A similar temporal separation was observed in the cassava group, where instars 5–9 separated from instars 11–21 along PC1 (40.28% variance) (Supplementary Figure S2B). Furthermore, pairwise Bray-Curtis distances within the wheat bran group were significantly greater than those within the cassava group (*p* < 0.001) (Figure 2B), indicating that diet type drives divergence in gut microbial community composition. Significant differences in inter-individual (beta) diversity were also observed across developmental stages within the wheat bran group (*p* = 0.013) (Figure 2C) and the cassava group (*p* = 0.044) (Figure 2D), confirming that gut microbial community structure was jointly shaped by larval developmental stage and dietary substrate, with dietary substrate exerting the stronger effect.

**Figure 2:**
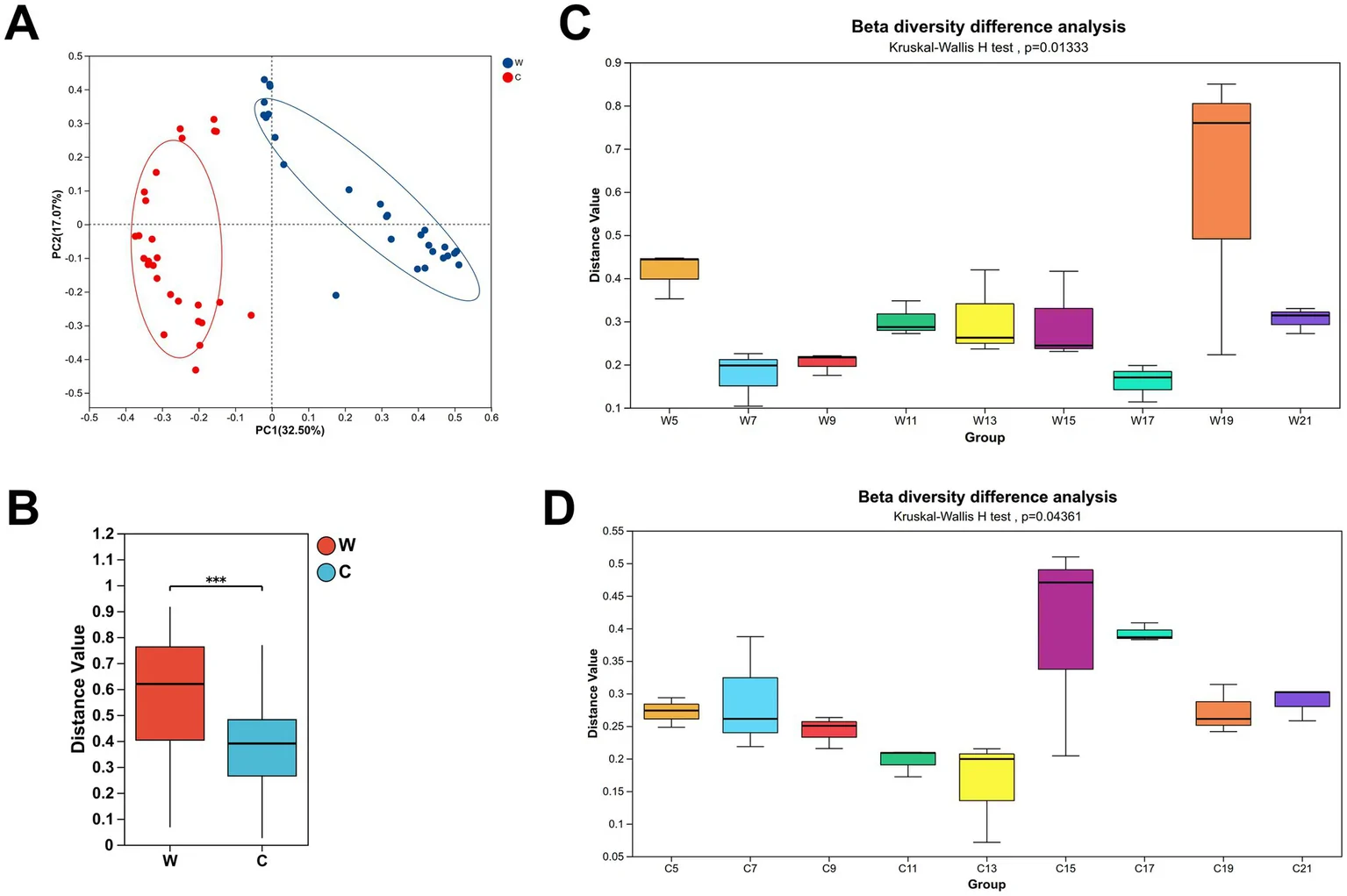
Beta diversity of intestinal bacterial microbiota in BSFL. **(A)** Principal coordinate analysis (PCoA) based on Bray-Curtis distances showing the structural dissimilarity of gut bacterial communities between wheat bran-fed (red) and cassava-fed (blue) larvae. **(B)** Comparison of Bray-Curtis pairwise distances between the wheat bran and cassava dietary groups. Statistical significance was determined by unpaired two-tailed Welch’s *t*-test (***p < 0.001). **(C,D)** Beta diversity dissimilarity (Bray-Curtis distances) among different larval instars within the wheat bran **(C)** and cassava **(D)** dietary groups. Statistical significance was determined by one-way ANOVA followed by Kruskal–Wallis H test (*p* < 0.05).

### 3.2 Dynamic changes of intestinal bacterial community in BSFL

At the phylum level, Proteobacteria, Firmicutes, Bacteroidota, and Actinobacteriota comprised the core microbiota across both diets (Figure 3A). However, their relative contributions differed markedly. Firmicutes was the most abundant phylum in the wheat bran group (52.34%), whereas Proteobacteria predominated in the cassava group (79.27%). Notably, Proteobacteria was the most abundant bacterial phylum in wheat bran-fed BSFL during early stages (instars 5–9), while Firmicutes predominated during instars 11–21 (Figure 3B). In the cassava-fed group, Proteobacteria declined progressively with instar but remained the most abundant phylum throughout development.

**Figure 3:**
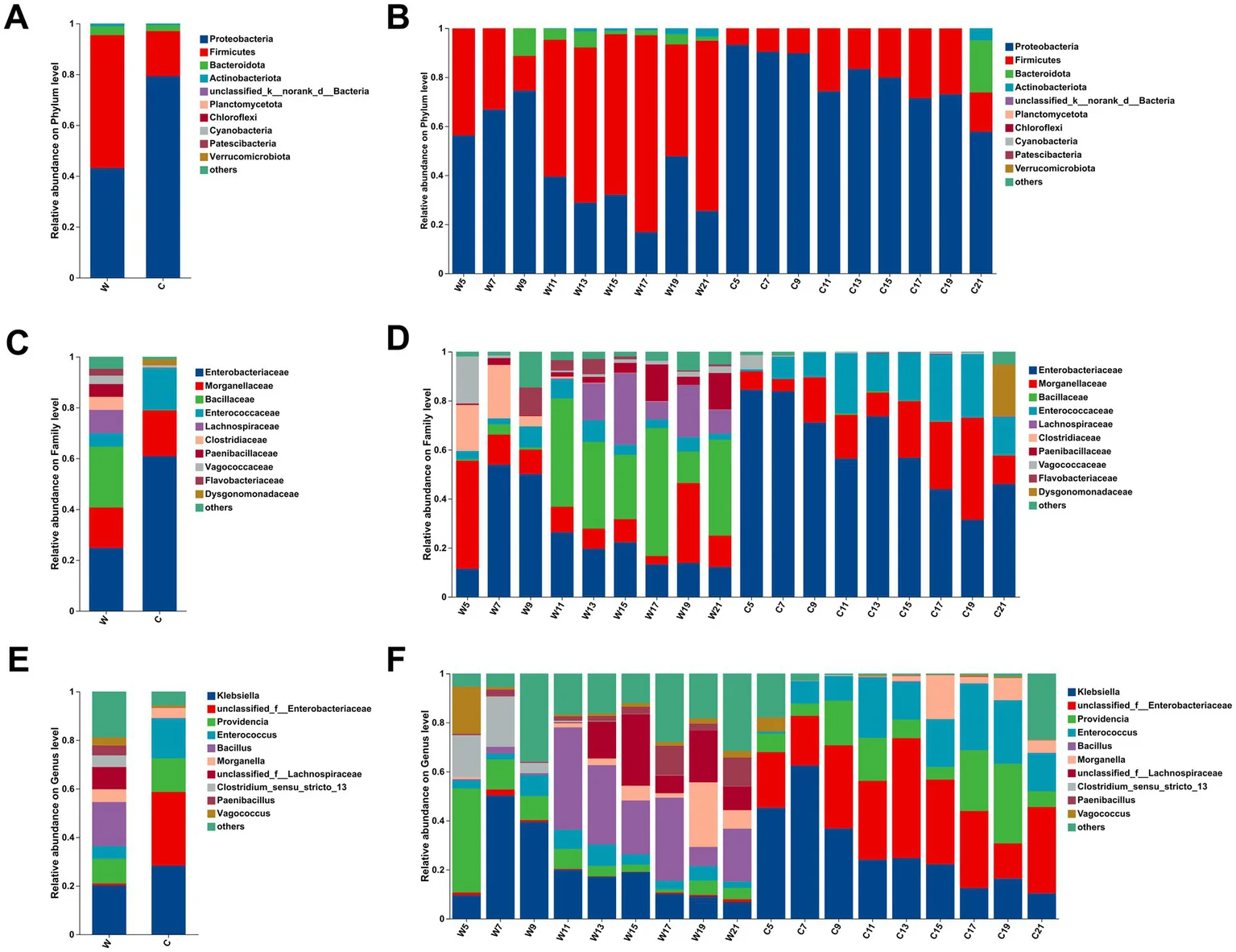
Composition of the intestinal bacterial microbiota in BSFL. **(A)** Overall microbial community structure across dietary treatments. **(B)** Phylum-level taxonomic composition across larval instars within the wheat bran and cassava diet groups. **(C,D)** Family-level composition across instars in the wheat bran group **(C)** and the cassava group **(D)**. **(E,F)** Genus-level composition across instars in the wheat bran group **(E)** and the cassava group **(F)**.

At the family level, the wheat bran group was dominated by Enterobacteriaceae (24.78%), Bacillaceae (23.93%), Morganellaceae (15.93%), Lachnospiraceae (9.22%), Enterococcaceae (5.24%), and Clostridiaceae (5.12%) (Figure 3C). Clostridiaceae was more abundant in the early stages (instars 5–9), whereas Bacillaceae and Lachnospiraceae became dominant in later stages (instars 11–21) (Figure 3D). In the cassava group, Enterobacteriaceae (60.87%) overwhelmingly dominated throughout development, followed by Morganellaceae (18.03%) and Enterococcaceae (16.48%). Notably, Dysgonomonadaceae increased abruptly at instar 21.

At the genus level, the wheat bran group harbored a diverse consortium including *Klebsiella* (20.17%), *Bacillus* (18.17%), *Providencia* (10.15%), and unclassified_f Lachnospiraceae (9.20%) (Figure 3E). The cassava group was dominated by unclassified_f_Enterobacteriaceae (30.41%), *Klebsiella* (28.28%), *Enterococcus* (16.48%), and *Providencia* (13.84%). In the wheat bran group, the relative abundance changes of *Clostridium_sensu_stricto_13,* Bacillaceae, and Lachnospiraceae were consistent with family-level trends (Figure 3F). In the cassava group, *Klebsiella* declined progressively with instar, while unclassified_f_Enterobacteriaceae, *Providencia*, and *Enterococcus* maintained dominance throughout development. Collectively, these results indicate that both diet and developmental stage shape the composition and abundance of the gut microbiota, with diet exerting the stronger effect.

### 3.3 Significant differences of intestinal microbiota in BSFL

To explore the differences in the bacterial communities between the two groups, we compared microbial communities across developmental stages. At the phylum level, cassava-fed larvae exhibited significant enrichment of Proteobacteria and marked reductions in Firmicutes and Bacteroidota relative to the wheat bran group (Figure 4A, Supplementary Figure S3A), indicating that starch-rich substrates favored copiotrophic Proteobacteria while suppressing fiber-degrading phyla. In the wheat bran group, Firmicutes, Proteobacteria, Bacteroidota, and Actinobacteriota each peaked at different instars (17th, 9th, 9th, and 21st instar, respectively) (Figure 4B), suggesting phased colonization in response to progressive fiber degradation. In the cassava group, Proteobacteria, Firmicutes, and Actinobacteriota peaked at the 5th, 19th, and 21st instar, respectively (Figure 4C). At the genus level, cassava-fed larvae were characterized by significantly higher abundances of *Klebsiella*, *Providencia*, *Enterococcus*, and *Enterobacter*, whereas wheat bran-fed larvae harbored greater abundances of *Bacillus*, *Clostridium_sensu_stricto_13*, *Paenibacillus*, *Vagococcus*, *Escherichia-Shigella*, and *Myroides* (Figure 4D). These results suggest that fiber-rich wheat bran favors degraders and fermenters, while starch-rich cassava promotes fast-growing, sugar-utilizing taxa. Within the wheat bran group, 10 genera showed significant shifts across instars, with most peaking during early development (5–9 d) (Figure 4E, Supplementary Figure S3B). In the cassava group, *Klebsiella* decreased progressively with larval age, whereas *Enterococcus* and *Providencia* dominated at the 17th and 19th instar, respectively (Figure 4F). The emergence of *Morganella* at the 15th instar and *Acinetobacter* at the 21st instar as stage-specific biomarkers (Supplementary Figure S3C) indicates that even under a simple starch diet, certain taxa are temporally constrained to specific developmental stages.

**Figure 4:**
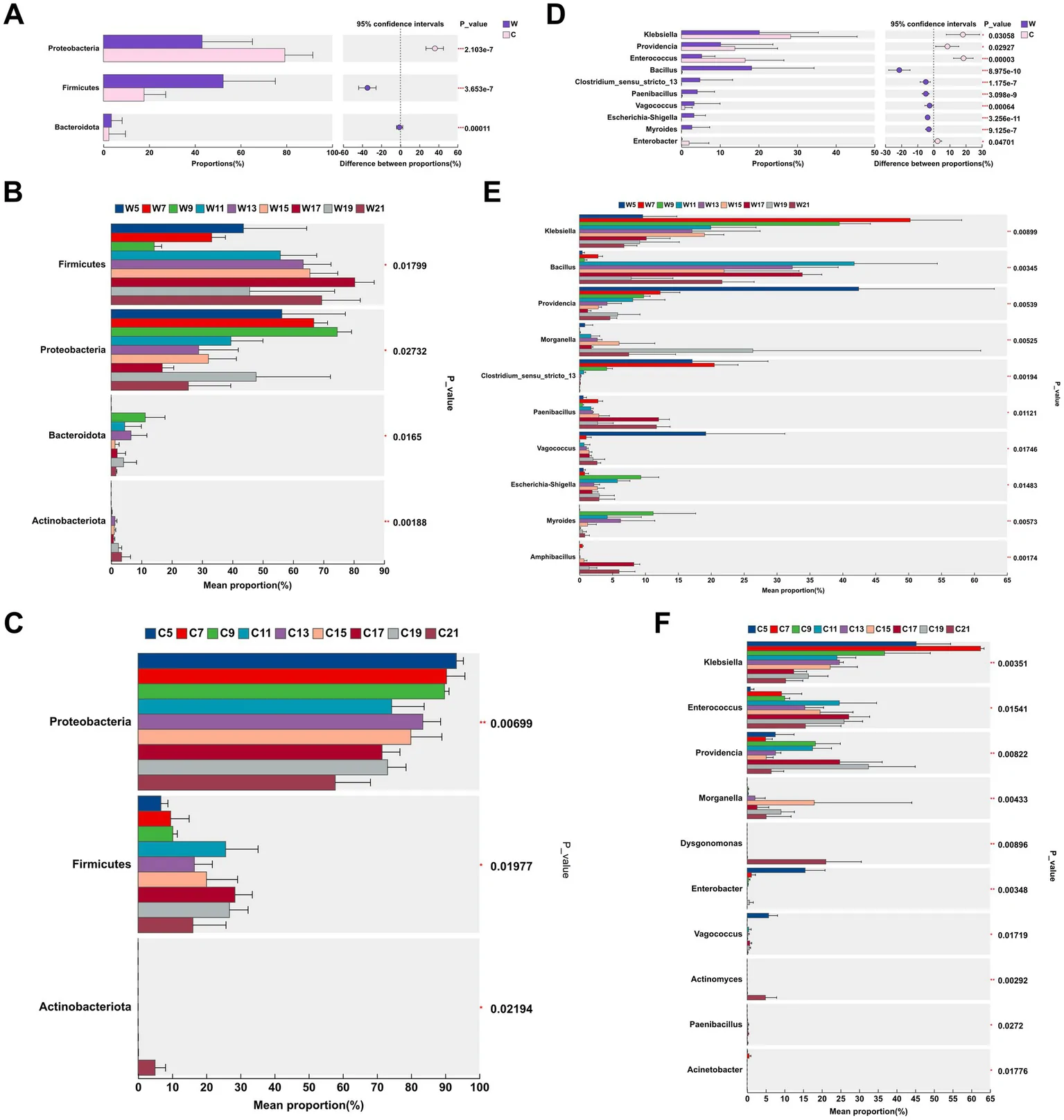
Statistically significant differences in intestinal microbiota composition of BSFL across dietary and developmental factors. **(A)** Differentially abundant bacterial taxa at the phylum level between the wheat bran and cassava diets. **(B,C)** Differentially abundant bacterial taxa at the phylum level across larval instars within the wheat bran group **(B)** and the cassava group **(C)**. **(D)** Differentially abundant bacterial taxa at the genus level between the wheat bran and cassava diets. **(E,F)** Differentially abundant bacterial taxa at the genus level across larval instars within the wheat bran group **(E)** and the cassava group **(F)**.

### 3.4 Diversity of intestinal bacterial communities across gut regions of BSFL

To investigate the divergence of the gut microbiota in different gut regions, we compared the foregut, midgut, and hindgut microbiota of instar 21 larvae fed either wheat bran or cassava. In the wheat bran group, the Shannon index of the hindgut was significantly lower than that of the foregut and midgut (Figure 5A), indicating reduced microbial diversity in the posterior compartment. In contrast, no significant differences in Shannon diversity were observed among the three gut regions in the cassava group (Figure 5B), suggesting a homogenously distributed microbiota under the starch-based diet. PCoA based on Bray-Curtis distances corroborated these results. In the wheat bran group, PC1 (52.47% variance) clearly separated the hindgut from the foregut and midgut, with the latter two forming a single cluster (Figure 5C). In the cassava group, the foregut and hindgut clustered together on PC1 (45.23% variance), while the midgut formed a separate group (Figure 5D), indicating that starch metabolism is primarily localized to the midgut. Pairwise Bray–Curtis distances among the three gut regions were not statistically significant in either the wheat bran group (*p* = 0.1133) or the cassava group (*p* = 0.3292), suggesting that gut region had a limited impact on community composition relative to dietary substrate.

**Figure 5:**
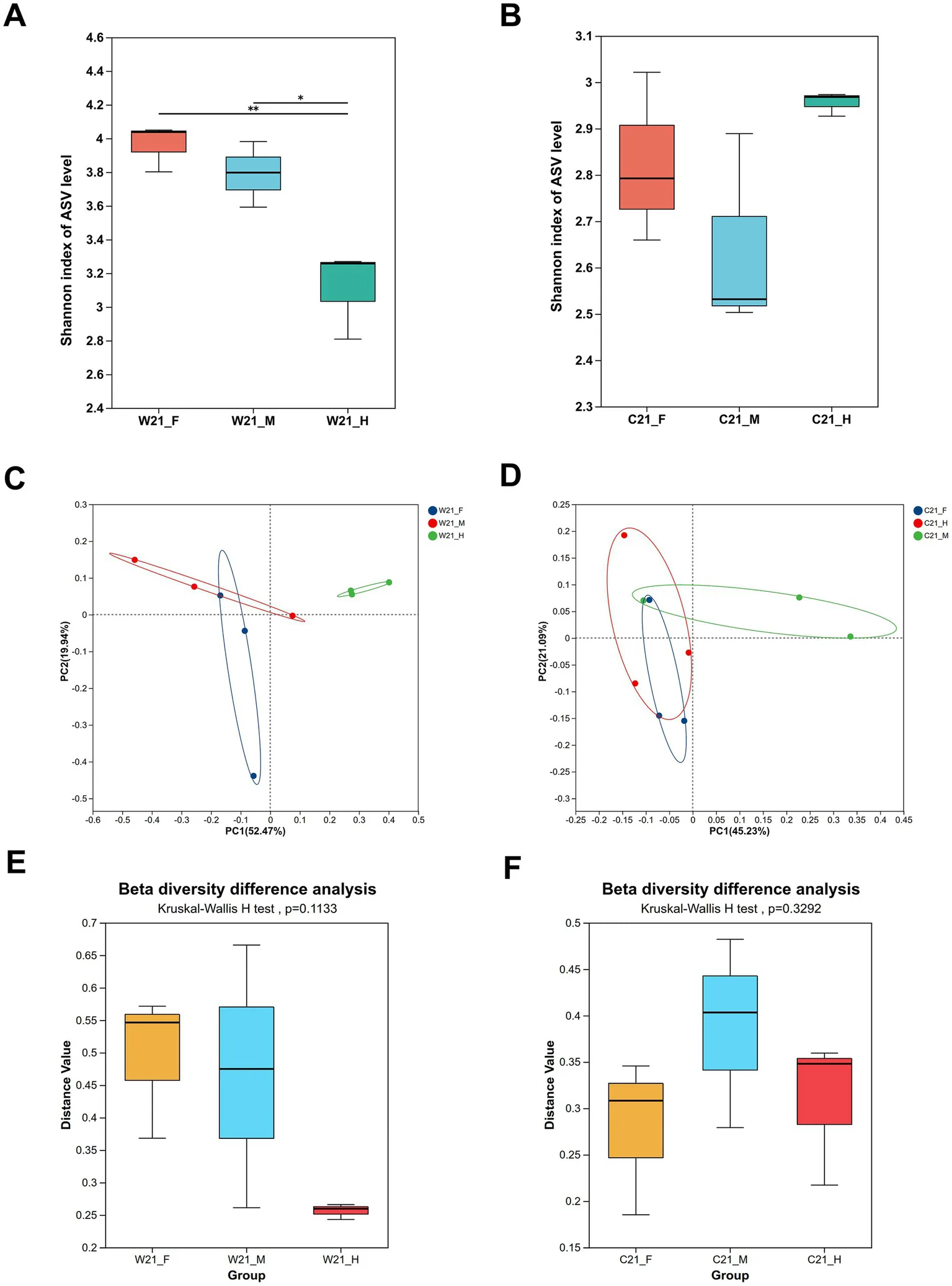
Diversity of intestinal bacterial communities in different intestinal regions (foregut, midgut, and hindgut) of BSFL. **(A,B)** Shannon diversity index of gut bacterial communities in the foregut, midgut, and hindgut of larvae fed wheat bran **(A)** and cassava **(B)**. Statistical significance was determined by one-way ANOVA followed by Tukey’s HSD *post-hoc* test, *p* < 0.05. *p < 0.05, ** *p* < 0.01. **(C,D)** PCoA based on Bray-Curtis distances showing the structural dissimilarity of gut bacterial communities across the foregut, midgut, and hindgut in the wheat bran **(C)** and cassava **(D)** groups. **(E,F)** Beta diversity dissimilarity (Bray-Curtis distances) among gut regions in the wheat bran **(E)** and cassava **(F)** groups. Statistical significance was determined by one-way ANOVA followed by Kruskal–Wallis H test (*p* < 0.05).

### 3.5 Composition of intestinal bacterial communities across gut regions of BSFL

Microbial community composition in different gut regions were presented in Figure 6. At the phylum level, wheat bran-fed larvae maintained a more balanced composition across gut regions, with Bacteroidota enriched in the foregut, Firmicutes dominating the midgut, and Proteobacteria prevailing in the hindgut (Figure 6A). In contrast, cassava-fed larvae exhibited a more uniform profile, with Proteobacteria, Firmicutes, and Actinobacteriota distributed relatively evenly (Figure 6B).

**Figure 6:**
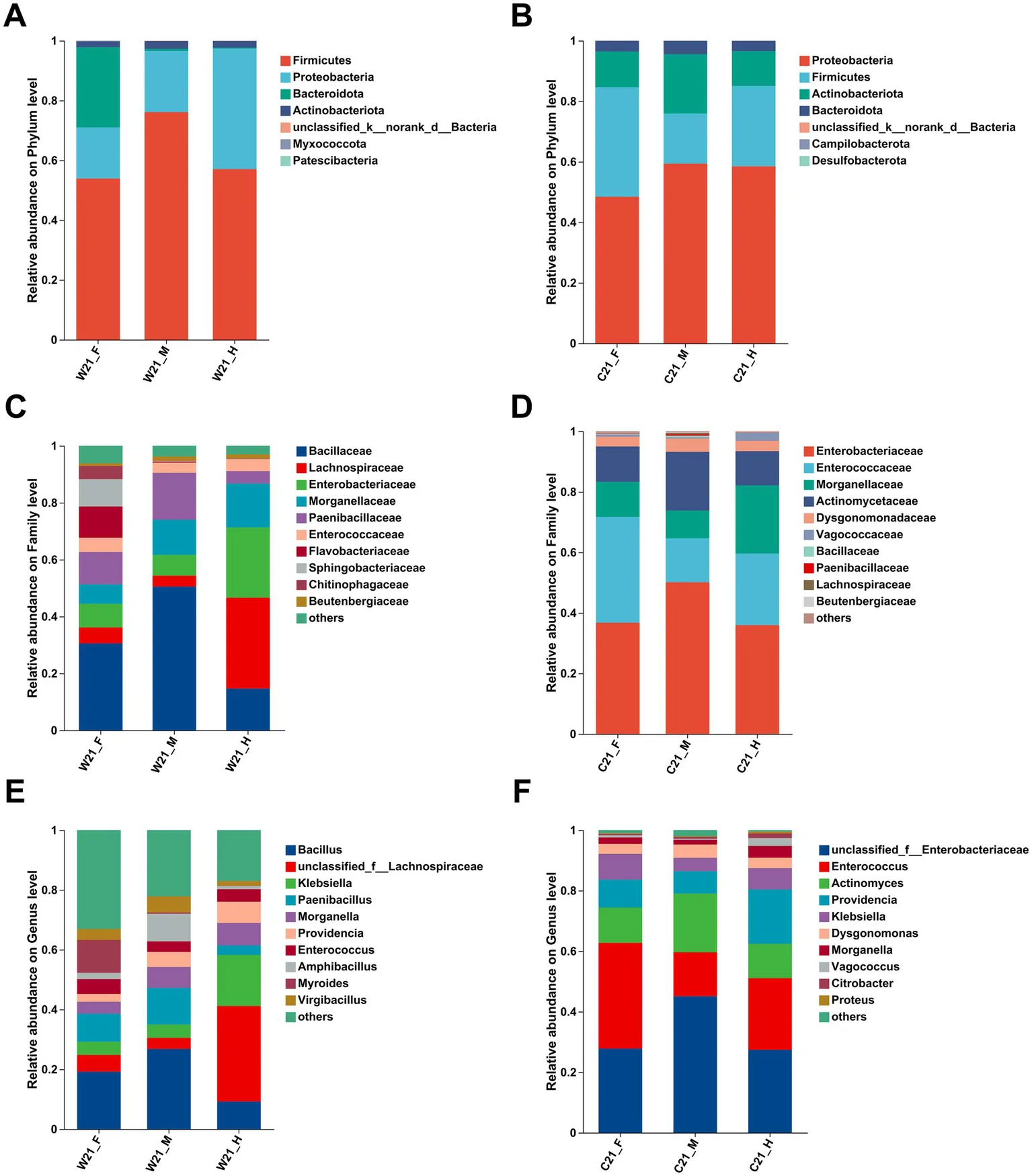
Microbial community composition across gut regions of BSFL. **(A,B)** Relative abundance of bacterial phyla in the foregut, midgut, and hindgut of larvae fed wheat bran **(A)** and cassava **(B)**. **(C,D)** Relative abundance of bacterial families across the three gut regions in the wheat bran **(C)** and cassava **(D)** groups. **(E,F)** Relative abundance of bacterial genera across the three gut regions in the wheat bran-fed **(E)** and cassava-fed **(F)** larvae.

At the family and genus levels, marked differences were observed between dietary groups. In the wheat bran group, Bacillaceae (primarily *Bacillus*) dominated both the foregut and midgut, whereas Lachnospiraceae (mainly unclassified_f_Lachnospiraceae) prevailed in the hindgut, with *Klebsiella* reaching its highest abundance in this posterior compartment (Figures 6C,E). This pattern indicates a multi-step degradation strategy for fibrous substrates. In contrast, cassava-fed larvae displayed a homogeneous distribution, with Enterobacteriaceae (primarily unclassified_f_Enterobacteriaceae) dominating across all three gut regions. Compartment-specific enrichments were limited to Enterococcaceae in the foregut, Actinomycetaceae in the midgut, and Morganellaceae in the hindgut at the family level (Figure 6D), and *Enterococcus* in the foregut, *Actinomyces* in the midgut, and *Providencia* in the hindgut at the genus level (Figure 6F). Collectively, these results demonstrate that the fibrous wheat bran diet promotes a more spatially structured community, whereas the starch-based cassava diet supports a homogeneous, midgut-centric community dominated by fast-growing fermentative taxa.

### 3.6 Significant differences of intestinal microbiota across gut regions of BSFL

In the wheat bran group, nine genera exhibited significant differential abundance among the foregut, midgut, and hindgut (Figure 7A). Notably, *Amphibacillus* was significantly enriched in the midgut, while *Myroides* was enriched in the foregut. In contrast, the cassava group displayed only two region-specific genera, *Enterococcus* and *Lachnoclostridium*, with *Enterococcus* consistently more abundant across all regions (Figure 7B).

**Figure 7:**
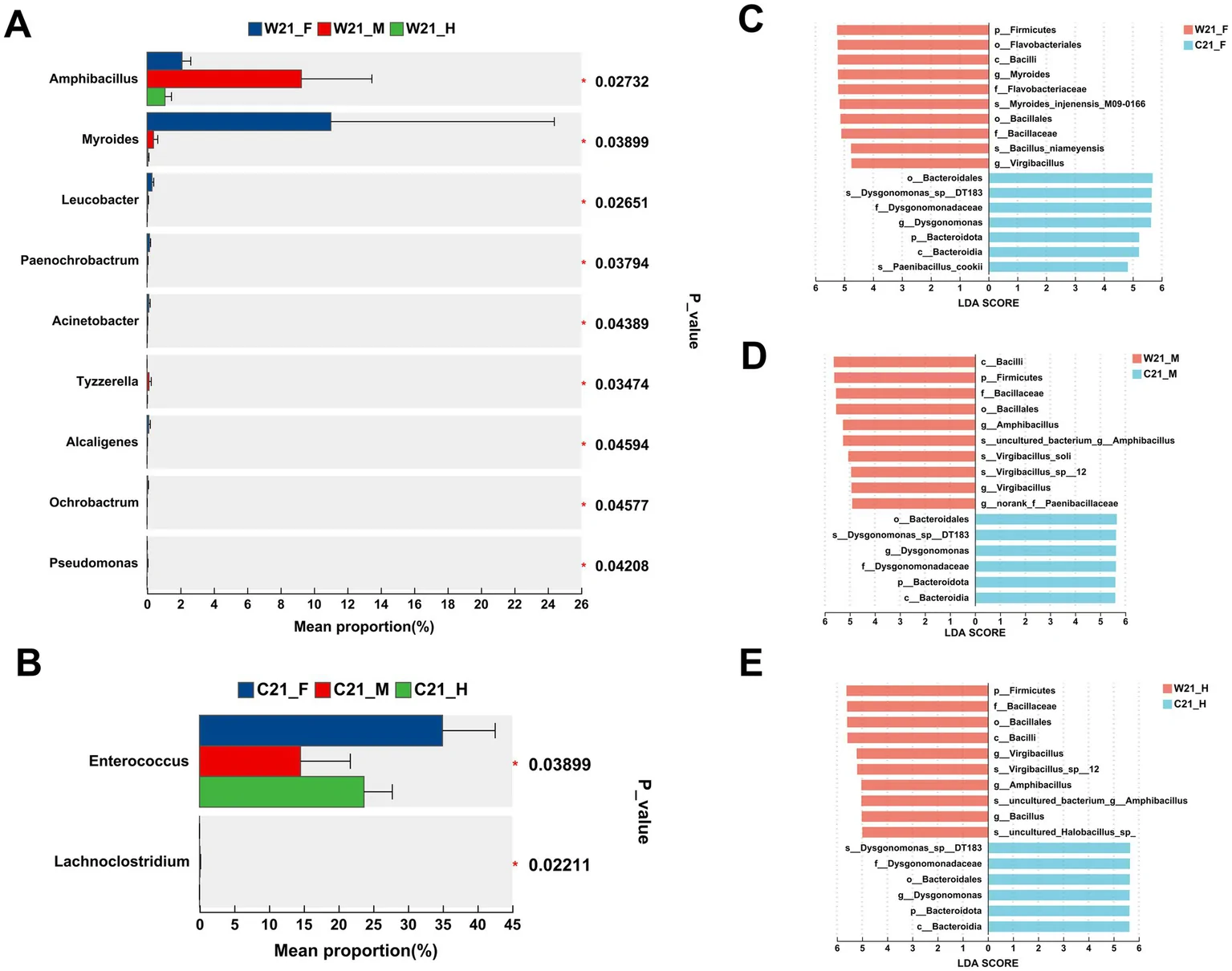
Significant differences in intestinal bacterial microbiota across gut regions of BSFL. **(A,B)** Differentially abundant bacterial genera among the foregut, midgut, and hindgut in the wheat bran **(A)** and cassava **(B)** groups. Significant differences across gut regions were determined by one-way ANOVA followed by Kruskal–Wallis H test (*p* < 0.05). **(C–E)** LEfSe analysis identifying the most differentially abundant bacterial taxa from phylum to genus level between wheat bran and cassava diets within the foregut **(C)**, midgut **(D)**, and hindgut **(E)** regions (LDA score threshold > 4). Bar length corresponds to the LDA score, reflecting the effect size of differential enrichment.

LEfSe analysis further revealed the discriminant taxa between dietary groups within each gut region (Figures 7C–E). In the foregut, wheat bran-fed larvae were enriched in p_Firmicutes, o_Flavobacteriales, c_Bacilli, and g_Myroides, whereas cassava-fed larvae were dominated by o_Bacteroidales, s_Dysgonomonas_sp_DT183, f_Dysgonomonadaceae, g_Dysgonomonas, and p_Bacteroidota (Figure 7C). In the midgut, the wheat bran group was enriched with c_Bacilli, p_Firmicutes, f_Bacillaceae, and g_Amphibacillus, while the cassava group maintained dominance of o_Bacteroidales, s_Dysgonomonas_sp_DT183, and g_Dysgonomonas (Figure 7D). In the hindgut, wheat bran-fed larvae were characterized by p_Firmicutes, f_Bacillaceae, g_Virgibacillus, and g_Amphibacillus, whereas cassava-fed larvae showed enrichment of s_Dysgonomonas_sp_DT183, f_Dysgonomonadaceae, o_Bacteroidales, and p_Bacteroidota (Figure 7E). Overall, the wheat bran diet fostered a more diverse and region-specific bacterial community, particularly enriching Firmicutes-affiliated taxa (e.g., Bacillaceae, *Amphibacillus*, *Virgibacillus*) likely involved in sequential fiber degradation. In contrast, the cassava diet consistently enriched *Dysgonomonas*-associated taxa across all gut regions, reflecting a more uniform and less differentiated community consistent with rapid starch metabolization in the anterior gut.

## 4 Discussion

This study investigated how dietary carbohydrate structure specifically fiber-rich wheat bran versus starch-rich cassava shapes the diversity, succession, and spatial organization of the gut microbiota in black soldier fly larvae (BSFL). Our results demonstrate that dietary substrate is the primary driver of gut community structure, exceeding the influence of developmental stage, and that carbohydrate complexity determines whether the microbiota undergoes progressive maturation and spatial compartmentalization or remains relatively homogeneous and dominated by a few fast-growing taxa.

The marked contrast in alpha diversity between the two diets supports the hypothesis that complex carbohydrates sustain a more diverse gut community. The wheat bran diet, rich in cellulose, hemicellulose, and other structural polysaccharides, requires a functionally diverse consortium of microorganisms for complete degradation. This is reflected in the significantly higher ACE, Chao, and Shannon indices in wheat bran-fed larvae and their progressive increase across instars. This pattern is consistent with a scenario involving progressive colonization and cooperative degradation; however, functional data are needed to confirm this interpretation. In contrast, the cassava diet, composed primarily of easily digestible starch, supported a less diverse community in which richness estimators (ACE and Chao) increased with age but the Shannon index did not. The Shannon index incorporates both species richness and evenness and is generally more influenced by abundant taxa, whereas ACE and Chao are richness estimators that rely on the frequency of rare taxa, particularly singletons and doubletons. Therefore, the observed increase in ACE and Chao without a corresponding increase in Shannon in the cassava group suggests that newly recruited taxa are primarily rare and do not substantially alter community evenness, which remains dominated by a few abundant starch-adapted fermenters. This pattern indicates that simple, readily metabolizable substrates favor a winner-takes-all competitive dynamic, whereas complex fiber promotes a more balanced and functionally redundant community.

The temporal succession observed in the wheat bran group from Proteobacteria-dominated early stages to Firmicutes-dominated later stages further supports a functional maturation of the fiber-degrading consortium. Proteobacteria, often dominant in early developmental stages of insects, are metabolically versatile and may act as early colonizers that initiate the breakdown of soluble components or partially hydrolyze complex polymers, thereby creating conditions favorable for subsequent colonizers (Auger et al., 2023; Zhao et al., 2017). Firmicutes, particularly members of Bacillaceae and Lachnospiraceae, are well known for producing extracellular cellulases, hemicellulases, and other glycoside hydrolases essential for degrading the polysaccharide matrix of wheat bran (Biddle et al., 2013; Wang et al., 2022). The sequential shift from Clostridiaceae in early stages to Bacillaceae and Lachnospiraceae in later stages is consistent with early colonizers preparing the substrate for more specialized degraders. In the cassava group, the persistent dominance of Proteobacteria (mainly Enterobacteriaceae) throughout development reflects the rapid and complete metabolization of starch in the anterior gut, leaving little recalcitrant substrate to drive a major phylum-level transition (Hildebrandt et al., 2009; Wastyk et al., 2021). Notably, Dysgonomonadaceae surged at instar 21 in the cassava group. Although Dysgonomonadaceae has been associated with polysaccharide degradation in other insect systems (Murakami et al., 2018; Owusu-Agyeman et al., 2022), its potential link to host lipid accumulation in cassava-fed larvae remains speculative and warrants further functional investigation.

A key finding of this study is the differential spatial organization of the gut microbiota induced by the two diets. In the wheat bran group, the hindgut exhibited significantly lower Shannon diversity than the anterior compartments, and PCoA clearly separated the hindgut from the foregut and midgut. Family- and genus-level composition further revealed a distinct turnover from Bacillaceae (foregut and midgut) to Lachnospiraceae (hindgut). This pattern points to a segmental processing strategy for fibrous substrates: Bacillaceae, known for producing extracellular amylases, cellulases, and proteases (Callegari et al., 2020), dominate the anterior gut where initial hydrolysis occurs, generating intermediate substrates that subsequently fuel the proliferation of fermentative Lachnospiraceae in the hindgut. Lachnospiraceae are renowned for producing short-chain fatty acids (SCFAs), which can serve as energy sources for the host (Yan and Zhou, 2026). The enrichment of Klebsiella in the hindgut may also contribute to nitrogen recycling or the degradation of specific fiber fractions, consistent with its recognition as a core member of the BSFL gut microbiota (Dar et al., 2018; Li et al., 2026; Seo et al., 2026).

In contrast, the cassava diet sustained a remarkably uniform community across all gut regions, with Enterobacteriaceae dominating throughout. The rapid metabolization of starch in the anterior gut likely leaves little recalcitrant substrate to drive longitudinal niche differentiation. The PCoA pattern, in which the midgut formed a separate cluster while foregut and hindgut clustered together, indicates that starch metabolism is predominantly localized to the midgut, with the anterior and posterior compartments exhibiting relatively similar communities. This midgut-centric metabolism is consistent with the rapid enzymatic hydrolysis of starch in the anterior digestive tract, leaving minimal substrate to drive longitudinal differentiation (Bruno et al., 2019). The absence of significant Shannon diversity differences across compartments and the non-significant beta diversity among gut regions quantitatively confirm limited spatial differentiation under the starch diet. These results imply that starch-rich substrates may not require spatial partitioning of digestive functions, potentially enabling rapid processing of readily available nutrients but offering fewer ecological niches for a diverse microbiota.

Differential abundance analyses further revealed that complex fiber promotes spatial niche partitioning to a greater extent than simple starch. The wheat bran diet was characterized by enrichment of Firmicutes-affiliated taxa, particularly Bacillaceae, Amphibacillus, and Virgibacillus, which are likely involved in sequential fiber degradation. In contrast, the cassava diet consistently enriched Dysgonomonas-associated taxa across all gut regions. Dysgonomonas is a genus within Bacteroidota associated with polysaccharide degradation, particularly hemicellulose and pectin, in diverse environments including insect guts and anaerobic digesters (Maus et al., 2020; Vera-Ponce de León et al., 2020). Its consistent dominance in cassava-fed larvae may reflect its ability to utilize starch-derived simple sugars or intermediate metabolites, positioning it as a key player in starch-based diets.

From an applied perspective, these findings suggest that fiber-rich agricultural residues may be particularly suitable for BSFL bioconversion because they promote a more diverse, functionally redundant, and spatially structured microbiota that could enhance substrate utilization efficiency and system stability. Conversely, starch-rich substrates such as cassava support a less diverse and more homogeneous community, which may increase the risk of proliferation of opportunistic pathogens. Indeed, the high relative abundance of Enterococcus and Providencia in cassava-fed larvae warrants attention from a biosafety perspective, as some members of these genera are opportunistic pathogens. While these genera are common in insect guts and may contribute to digestion, their dominance under starch-rich conditions highlights the need for careful substrate selection and post-harvest processing when BSFL are used as animal feed.

### 4.1 Limitations

Several limitations of this study should be acknowledged. First, our analyses are based on 16S rRNA gene sequencing, which provides taxonomic resolution but limited functional inference. Metagenomic or metatranscriptomic approaches would be valuable to confirm the functional roles of key taxa. Second, the correlation between Dysgonomonadaceae enrichment and lipid accumulation at 21 d requires direct metabolic profiling to establish causality. Third, potential contamination from the rearing environment cannot be entirely excluded, although containers were sterilized and the rearing room was maintained under positive pressure with HEPA-filtered air. Future studies using gnotobiotic or axenic rearing systems would help disentangle host- and environment-derived microbial contributions. Finally, our findings were obtained under controlled laboratory conditions; validation under large-scale industrial rearing conditions is necessary to ensure practical applicability.

## 5 Conclusion

In conclusion, this study provides a comprehensive characterization of the gut microbiota of BSFL reared on wheat bran and cassava diets, revealing that dietary carbohydrate structure fundamentally dictates the assembly, succession, and spatial organization of the microbial community. Dietary substrate is the dominant factor structuring the BSFL gut microbiota, with the fiber-rich wheat bran diet supporting significantly higher diversity, richness, and evenness than the starch-rich cassava diet. The wheat bran diet promotes a progressive maturation of the microbiota from Proteobacteria-dominated pioneers to Firmicutes-dominated specialists, whereas the cassava diet sustains a relatively static community dominated by Enterobacteriaceae throughout development. The fibrous wheat bran diet drives pronounced anterior–posterior differentiation (from Bacillaceae in the foregut/midgut to Lachnospiraceae in the hindgut), reflecting a segmental processing strategy, while the starch-based cassava diet supports a homogeneous, midgut-centric community with limited spatial partitioning. Taken together, our results establish a framework for understanding substrate-microbiome interactions in BSFL and provide a basis for rational optimization of feeding strategies in large-scale waste bioconversion systems. Future research integrating functional genomics, gnotobiotic experimentation, and confirmatory experiment will further advance our ability to harness the BSFL gut microbiome for sustainable organic waste valorization.

## Data Availability

The datasets presented in this study are not readily available because the raw sequencing data are associated with an unpublished intellectual property, and public release at this stage could compromise the protection of these rights. Requests to access the datasets should be directed to the corresponding author, Dr. Shaojuan Liu, at lishaojuan@scau.edu.cn.
