# Supplementary material for: Dietary carbohydrate structure affects the gut microbial diversity, succession, and compartmentalization in black soldier fly larvae
Source: Front Microbiol. 2026 Oct 29;17:1956925. doi: 10.3389/fmicb.2026.1956925 (PMC13754821; doi:10.3389/fmicb.2026.1956925)
Supplement: Supplementary file 1 [file Supplementary_file_1.DOCX]

**Supplementary materials**

**Table S1. The nutritional components of wheat bran diet and cassava diet.**

| Components (% dry matter) | Wheat bran | Cassava |
| --- | --- | --- |
| Dry matter | 89.2 | 87.5 |
| Crude protein | 15.8 | 2.1 |
| Crude fat | 4.2 | 0.8 |
| Crude fiber | 42.5 | 3.2 |
| Nitrogen-free extract | 32.1 | 82.4 |
| Ash | 5.4 | 1.5 |

**Figure S1. Rank-abundance curves comparing microbial community composition across samples from wheat bran- and cassava-based diets.**

**Figure S2. PCoA plots based on Bray–Curtis** **dissimilarity metrics for samples from BSFL fed either (A) wheat bran or (B) cassava diets across developmental instars.**

**Figure S3. LEfSe analysis of the most differentially abundant bacterial taxa of BSFL**. (A) Taxa exhibiting significant differential abundance between the wheat bran and cassava diets, spanning taxonomic levels from phylum to genus. (B) Differentially abundant bacterial taxa identified within the wheat bran diet group, and (C) within the cassava diet group, across larval developmental instars (LDA score threshold > 4). Bar length corresponds to the LDA score, reflecting the effect size of differential enrichment.
